# Poor Sleep Quality in Nurses Working or Having Worked Night Shifts: A Cross-Sectional Study

**DOI:** 10.3389/fnins.2021.638973

**Published:** 2021-08-03

**Authors:** Qiao Huang, Chong Tian, Xian-Tao Zeng

**Affiliations:** ^1^Center for Evidence-Based and Translational Medicine, Zhongnan Hospital of Wuhan University, Wuhan, China; ^2^Department of Evidence-Based Medicine and Clinical Epidemiology, Second School of Clinical Medicine, Wuhan University, Wuhan, China; ^3^School of Nursing, Tongji Medical College, Huazhong University of Science and Technology, Wuhan, China; ^4^Department of Urology, Zhongnan Hospital of Wuhan University, Wuhan, China

**Keywords:** clinical nurse, night shift, sleepiness, poor sleep quality, S-shaped relationship

## Abstract

Night shifts are part of clinical care. It is unclear whether poor sleep quality of nurses working both consecutive night shifts and day shifts after quitting night shifts is common. In this cross-sectional study, Pittsburgh Sleep Quality Index (PSQI) was used to assess sleep quality as study outcome. Univariable and multivariable linear and logistic regressions were performed to compare PSQI score and prevalence of poor sleep quality between 512 nurses currently working consecutive night shifts and 174 nurses having worked night shifts in the past. The prevalence of poor sleep quality was 62.11% in nurses working consecutive night shifts and 55.75% in nurses having worked night shifts before. In multivariable regressions with adjustment for potential confounders, compared with nurses working consecutive night shifts, nurses having worked past night shifts reported decreased PSQI score [mean difference: −0.82 (95% CI: −1.27 to −0.38, *p* < 0.001)] and lower poor sleep quality [odds ratio (OR): 0.49 (95% CI: 0.29 to 0.80, *p* = 0.005)]. In nurses working consecutive night shifts, a rising curve that plateaued at the end was observed between years of consecutive night shifts and PSQI score, *p* = 0.004. To explore the change in PSQI score after quitting night shift, we constructed a hypothetical prospective cohort from the cross-sectional data. Here, 98 pairs of nurses with consecutive and past night shifts were matched for the number of night shift years, religion, marital status, living condition, hypertension, and hyperlipidemia. In each pair, a hypothetical change in PSQI score was calculated between the two types of nurses and hypothetical years after quitting night shifts was obtained from the matched nurse with past night shifts. A U-shaped curve between change in PSQI and years after quitting night shifts was observed, *p* = 0.007. The rising curve and U-shaped curve together formed an S-shaped curve, which mapped the change in sleep quality. These results based on the hypothetical cohort constructed from cross-sectional data suggested the presence of persistent poor sleep quality in night shift nurses. Also, we support early and continuous sleep hygiene education and reflection for an optimal strategy for when to cease working night shifts with regard to sleep-related problems.

## Introduction

Suprachiasmatic nuclei, which control circadian rhythm, are sensitive to signals of light and darkness (Welsh et al., 2010). Night shift work is likely to disrupt circadian rhythms. A review with 38 meta-analyses and 24 systematic reviews has presented the impact of shift work on sleep, mainly manifested as acute sleep loss (Kecklund and Axelsson, 2016). Meanwhile, disturbed circadian rhythm leads to change in ghrelin, leptin, insulin, cortisol, and melatonin levels among night shift workers, causing deterioration in sleep quality (Ulhôa et al., 2015).

To provide professional patient care 24/7, clinical healthcare workers need to participate in a rotating schedule, which requires shifts to rotate through prescribed intervals between day, evening, night, and early morning (Canuto et al., 2013). A higher prevalence of sleep problems was reported in shift-working nurses compared to the general population (Sun et al., 2019). A large survey showed that the prevalence of symptoms of shift work disorder (such as difficulties with sleeping, excessive sleepiness, and insomnia) in night shift nurses was about 40% (Flo et al., 2012). Night shift work in healthcare workers increased the risk of sleep disorders, insomnia, and excessive daytime sleepiness (Booker et al., 2018; Rosa et al., 2019). Family factors, such as being married, further increased the risk of night shift-related sleep impairment due to increased family responsibilities (Booker et al., 2018). The majority of nurses were female; a study of female nurses found that psychological distress, especially depression, was associated with sleep disturbance (Chueh et al., 2021). Meanwhile, menopause-related symptoms also greatly influenced sleep quality (Pien et al., 2008). Hypertension, hyperlipidemia, diabetes, and alcohol consumption were associated with poor sleep quality (Chiang et al., 2018; Wang et al., 2020; Zheng et al., 2020).

According to a large survey conducted by the Chinese National Centre for Nursing Care Quality Control in 2017, one nurse was responsible for eight patients during the day and 23 patients at night (Shen et al., 2020). Based on the classification of Chinese hospitals, a tertiary and graded A hospital would service a large number of patients; however, the bed-to-nurse ratio was not satisfactory (Bin-bin et al., 2018). Nursing shortages and heavy clinical nursing workload force almost all nurses to undertake night shift work from the beginning of their nursing careers. Frequent night shifts lead to disturbance in sleep mechanisms and make it difficult for clinical nurses to adapt. After years of consecutive night shifts, nursing managers may arrange for older nurses to work only day shifts as the night shifts could be filled by fresh nurses. When this happens, older nurses have to readapt to a normal day shift pattern. Former shift nurses, current shift nurses, and non-shifting nurses reported poor sleep quality (Rotenberg et al., 2011; McDowall et al., 2017). Poor sleep quality in former shift nurses might be due to the accumulated exposure to night shift, increasing the risk of developing chronic sleep problems (Ingre and Akerstedt, 2004). Meanwhile, poor sleep quality in current shift- and non-shift-working nurses might be due to high levels of burnout and stress.

Addressing how clinical nurses’ sleep quality changes during consecutive night shifts and after quitting is essential to help establish optimal sleep improvement strategies to minimize the impact of night shifts on sleep quality. There have been many studies on the impact of night shifts on nurses’ sleep quality; however, there was little evidence of change in nurses’ sleep quality during and/or after night shift work in long-term observation studies. In this cross-sectional study, clinical Nurses who were on constant and Consecutive Night Shifts (NCNS) and Nurses who had Past Night Shifts (NPNS) were recruited. Firstly, we assessed differences in sleep quality between NCNS and NPNS. Secondly, we explored the possible relationship between sleep quality and years of consecutive night shifts in the NCNS group. Finally, we constructed a hypothetical prospective cohort from the cross-sectional data and explored the potential relationship between a hypothetical change in sleep quality and hypothetical years after quitting night shift.

## Materials and Methods

### Study Design, Setting, Participants

From April to June 2016, a multicenter, cross-sectional, hospital-based survey was conducted in four tertiary and graded A hospitals in Wuhan city, China. When a clinical nurse’s rotating schedule includes or partially covers a period from 00:01 to 06:00, that nurse is defined as working on a rotating night shift. Two types of clinical nurses were considered as eligible participants for inclusion: (1) NCNS, the clinical nurses who had been working on rotating night shifts from the start of their nursing career until the survey month; (2) NPNS, those clinical nurses who had been working night shifts in the past but had quit night shifts before the survey month. Since the number of male nurses was low in China, they were excluded to avoid gender bias. Participants with missing Pittsburgh Sleep Quality Index (PSQI) score were also excluded.

### Data Collection, Study Factors, and Outcome

A self-administered questionnaire was designed and reviewed to collect participant-reported data. It consisted of four parts to achieve the purpose of our study: (1) basic characteristics, (2) social–psychological scales, (3) information regarding rotating night shift work related, and (4) sleep quality. Four directors of nursing departments were contacted in advance; the head nurse in each department was responsible for distributing and collecting the printed questionnaires.

Basic characteristics collected were participants’ social–demographical data, including age, religion (yes or no), educational level, marital status, living environment, family annual income, current smoking status, and current alcohol consumption. Participants who reported smoking at least one cigarette per day for more than 6 months were defined as current smokers. Participants were identified as current alcohol consumers when they reported consuming alcohol at least once per week for more than 6 months. Hypertension, hyperlipidemia, diabetes, and menopause status were also collected.

Negative psychological states were measured using two scales: Irritability, Depression and Anxiety (IDA) Scale and Perceived Stress Scale (PSS). IDA Scale is a self-assessment tool to assess depression level, anxiety level, and inward and outward irritability (Snaith et al., 1978). The 18-item validated Chinese version was used (Yuan et al., 2002). PSS is another validated instrument to measure non-specific perceived stress (Cohen et al., 1983). A Simplified Chinese version of PSS-14, utilized for medical residents, was adopted in our study (Shi et al., 2019).

Information on rotating night shift was collected. The status of rotating night shifts (current night shifts vs. past night shifts) was considered our key study factor. NCNS were asked to report duration (years) of rotating night shifts, while NPNS were asked to report both duration of rotating night shift (years) and total duration of nursing work (years). Sleep quality as the study outcome was measured using a self-administered and standardized PSQI questionnaire, which was reliable and valid in assessing sleep problems for multiple populations (Buysse et al., 1989). It consisted of 19 items and seven components (quality, latency, duration, efficiency, disturbances, use of sleep medication, and daytime dysfunction). A global score greater than five indicated poor sleep quality. A systematic review showed that PSQI was a common instrument to measure shift-working nurses’ sleep quality (Kang et al., 2020). In this study, a validated Chinese version was used (Tsai et al., 2005).

After collection of the printed questionnaire, a unique reference number was allocated for each questionnaire for de-identification and confidentiality. Data were entered into EpiData (version 3.1, EpiData Association, Denmark) by two independent researchers; two completed data sets were compared by a third researcher to eliminate any inconsistency.

### Statistical Analysis

Categorical variables were described using frequencies and percentages and compared using chi-square test or Fisher’s exact test. Continuous variables were expressed as median with interquartile ranges (IQRs) and were compared using the Wilcoxon rank-sum test because of non-normal distribution.

Potential confounders influencing sleep quality had been determined in the introduction. Firstly, to explore the independent association between night shift status and sleep quality, we performed hierarchical multivariable linear regressions using PSQI score as the dependent variable to estimate the difference in PSQI score between two groups; meanwhile, we conducted hierarchical multivariable logistic regression using dichotomous PSQI (PSQI score > 5) as the dependent variable to explore the effect of night shift status on the prevalence of poor sleep quality. In model 1, only the night shift status of interest (past vs. current) was included. In model 2, basic characteristics were adjusted. Because there was a strong collinearity between age and duration of total nursing work (Spearman’s correlation coefficient = 0.93), duration of total nursing work was kept in model 2. Few nurses in the two groups had technical secondary or graduate degrees, were current cigarette smokers, had diabetes, or were undergoing menopause, so these variables were not included. In model 3, IDA and PSS scores were further adjusted. All related variables were included in model 3; multicollinearity was checked using variance inflation factor (VIF), and a VIF < 10 for each variable was acceptable. Normality of residuals was checked using a histogram, and heteroscedasticity was checked using the White test for linear regression. Also, outliers were checked based on a histogram of residuals. For logistic regression, pseudo R-squared was estimated and goodness of fit was checked using Hosmer–Lemeshow test. In model 4, covariate adjustment using propensity score was performed to control for selection bias and potential overfitting (Elze et al., 2017). Propensity scores were calculated by logistic regression to represent the conditional probability of night shift status based on confounders in model 3.

Secondly, to explore the non-linear relationship between duration of consecutive night shifts and sleep quality in NCNS, we conducted a multiple linear regression with restricted cubic spline (RCS). In NCNS, years of consecutive night shifts were equal to years of total nursing work. Years of consecutive night shifts were used as the timescale in RCS, and the number of knots was determined based on the Akaike information criterion. Other confounders in the aforementioned model 3 were adjusted.

**FIGURE 1:**
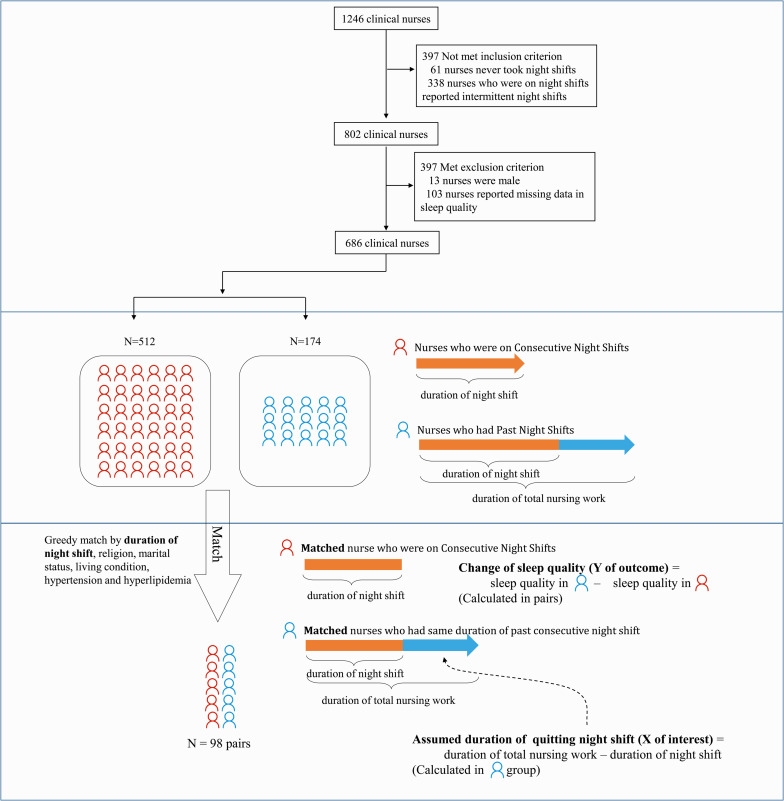
Framework for constructing a hypothetical prospective cohort using greedy match.

Lastly, to assess the relationship between years after quitting night shift work and the change of sleep quality, we constructed a hypothetical prospective cohort based on the cross-sectional data (Figure 1). NCNS were matched to NPNS with the same duration of night shift, religion, marital status, living condition, hypertension, and hyperlipidemia using the greedy-match method. Annual family income generally grew with working duration and age, so it was not used in matching. In each pair, matched NPNS and NCNS shared the same years of consecutive night shifts; the NPNS unit could be thought of as the NCNS unit who had quit night shift for years. Time since quitting night shift was calculated in NPNS (duration of nursing work minus duration of night shift), and change of sleep quality after quitting night shift was calculated using the difference in PSQI between two units.

The data were analyzed by SAS software (Version 9.4 TS1M6, SAS Institute Inc., Cary, NC) and R software (The R Foundation for Statistical Computing, Vienna, Austria) with rms package. Two-sided *p*-values were reported for hypothetic tests.

## Results

A total of 1,246 nurses gave informed consent and filled out the questionnaires. Among them, 61 nurses had never worked night shifts and 338 nurses reported working intermittent night shifts. Then, 588 NCNS and 214 NPNS were included. Also, 13 male nurses and 103 participants with missing sleep quality data were excluded. Finally, 686 nurses were included for analysis, including 512 NCNS and 174 NPNS (Figure 1). Median years of consecutive and past night shifts were 4 years and 11 years, respectively. Age, duration of nursing work, and rate of being married in NCNS were significantly lower than those in NPNS. In addition, 88.44% NPNS and 49.22% NCNS lived with parents and child. The rates of current smoking, alcohol consumption, hypertension, diabetes, and menopause were low in both groups, and there were no statistical differences. Hyperlipidemia in NPNS was statistically higher than that in NCNS, 13.22% vs. 4.69%. More details of characteristics were presented in Table 1. Neither the four components of IDA nor the two components of PSS showed any statistical differences. See Supplementary Table 1.

**TABLE 1:** Basic characteristics of nurses working consecutive night shifts and nurses having worked past night shifts.

Characteristics	Nurses with consecutive night shifts (*n* = 512)	Nurses with past night shifts (*n* = 174)	p
Duration of night shifts, years, median (Q1, Q3)	4.00 (3.00, 9.00)	–	–
Duration of night shifts in the past, years, median (Q1, Q3)	–	11.00 (5.00, 17.00)	–
Duration of total nursing work, years, median (Q1, Q3)	4.00 (3.00, 9.00)	14.50 (6.50, 24.00)	<0.001
Age, years, median (Q1, Q3)	27.00 (25.00, 31.00)	35.00 (29.00, 43.00)	<0.001
Any religion, *n* (%)	15 (2.98%)	7 (4.05%)	0.43
Educational level, *n* (%)			0.05
Technical secondary degree	2 (0.39%)	3 (1.75%)	
Junior or undergraduate degree	500 (97.66%)	161 (94.15%)	
Graduate degree	10 (1.95%)	7 (4.09%)	
Married, *n* (%)	252 (49.22%)	148 (85.06%)	<0.001
Living environment, *n* (%)			<0.001
Live alone	137 (26.92%)	11 (6.36%)	
Live with parents or children	295 (57.96%)	153 (88.44%)	
Other	77 (15.13%)	9 (5.20%)	
Annual family income level, *n* (%)			<0.001
Less than 50,000 yuan	99 (19.84%)	17 (10.00%)	
50,001∼100, 000 yuan	235 (47.09%)	60 (35.29%)	
100,001∼200, 000 yuan	138 (27.66%)	74 (43.53%)	
More than 200, 000 yuan	27 (5.41%)	19 (11.18%)	
Current alcohol consumer, *n* (%)	42 (8.20%)	21 (12.07%)	0.13
Current cigarette smoker, *n* (%)	3 (0.37%)	0 (0.00%)	1.00
Hypertension, *n* (%)	9 (1.76%)	8 (4.60%)	0.07
Hyperlipidemia, *n* (%)	24 (4.69%)	23 (13.22%)	<0.001
Diabetes, *n* (%)	4 (0.78%)	1 (0.57%)	1.00
Menopause, *n* (%)	2 (0.49%)	3 (1.89%)	0.27
Pittsburgh Sleep Quality Index (PSQI)			
PSQI global score, median (Q1, Q3)	6.00 (5.00, 8.00)	6.00 (4.00, 8.00)	0.04
Poor sleep quality, *n* (%)^*a*^	318 (62.11%)	97 (55.75%)	0.14

*^*a*^PSQI score > 5. PSQI, Pittsburgh Sleep Quality Index.*

Sleep quality measured by PSQI was presented in Table 1. The median of PSQI scores was 6 in both NCNS and NPNS, but a statistical difference existed between the two groups. Here, 62.11% NCNS and 55.75% NPNS reported poor sleep quality. Examination of individual PSQI components gave further insights (Supplementary Table 1). NCNS recorded higher scores in subjective sleep quality and sleep latency than NPNS, *p* = 0.03 and 0.04, respectively. While a higher score in the use of sleep medication was observed in NPNS, *p* = 0.03. There was no statistical difference in sleep duration, habitual sleep efficiency, sleep disturbance, or daytime dysfunction between the two groups.

In Table 2, for linear regression in model 3, *R*^2^ was 0.33, the maximum value of VIF was 2.39, which indicated weak multicollinearity; an approximately normal distribution of residuals was observed, and the *p*-value for White test was 0.11, indicating homogeneity of variance of the residuals. For logistic regressions in model 3, pseudo *R*^2^ was 0.29, and the *p*-value for Hosmer–Lemeshow test was 0.58. Estimates and 95% confidence intervals (CIs) were presented. In model 1, without adjustment, neither of the two regressions showed significant associations, *p* = 0.11 and 0.15. After adjustment for basic characteristics, NPNS showed a statistically decreased PSQI score, and the adjusted coefficient was −0.91 (95% CI: −1.43 to −0.40) using raw PSQI score as the dependent variable; the adjusted OR was 0.52 (95% CI: 0.33–0.81) using dichotomized PSQI as the dependent variable. With further adjustment for irritability, depression, anxiety, and stress in model 3, the adjusted coefficient was −0.82 (95% CI: −1.27 to −0.38), and the adjusted OR was 0.49 (95% CI: 0.29 to 0.80, *p* = 0.005). Using propensity score adjustment as the sensitivity analysis, the association did not change appreciably, suggesting that the estimates were robust.

**TABLE 2:** Linear and logistic regressions to explore the difference in sleep quality between nurses working consecutive night shifts and nurses having worked past night shifts.

Past vs. consecutive	PSQI global score (raw)		Poor sleep quality (PSQI > 5)	
	Coefficient (95% CI)	*p*	OR (95% CI)	*p*
Model 1	−0.35 (−0.79, 0.08)	0.11	0.77 (0.54, 1.10)	0.15
Model 2	−0.91 (−1.43, −0.40)	0.001	0.52 (0.33, 0.81)	0.004
Model 3	−0.82 (−1.27, −0.38)	<0.001	0.49 (0.29, 0.80)	0.005
Model 4	−0.77 (−1.31, −0.23)	0.005	0.59 (0.38, 0.92)	0.02

*PSQI, Pittsburgh Sleep Quality Index; CI, confidence interval; OR, odds ratio. Model 1, no adjustment; Model 2, adjusted for religion, marital status, living condition, income, duration of nursing work, alcohol consumption, hypertension, and hyperlipidemia; Model 3, further adjusted for irritability, depression, and anxiety score and perceived stress score based on model 2; Model 4, adjusted for propensity score calculated based on covariates in model 3.*

**FIGURE 2:**
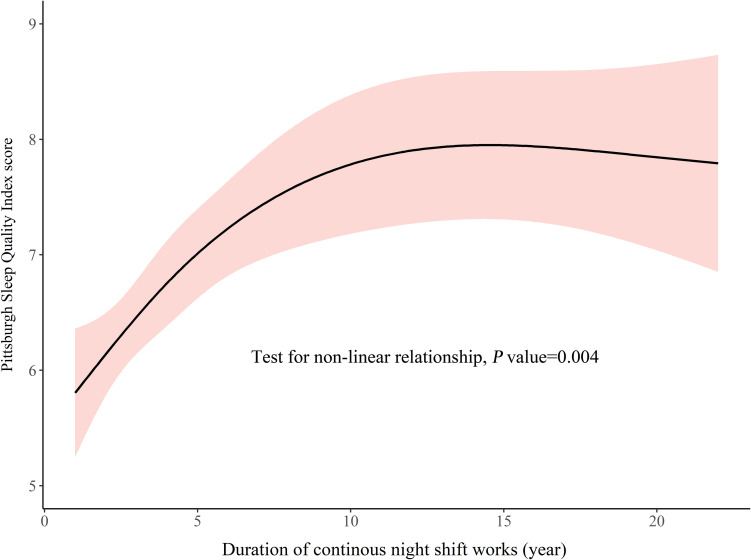
Change of sleep quality during the duration of consecutive night shifts.

In the NCNS group, the duration of consecutive night shifts and PSQI demonstrated a rising curve that plateaued at the end, *p* = 0.004, see Figure 2. From the early stage of night shifts (≥1 year), clinical nurses had presented poor sleep quality (>5). Over about 12 years of consecutive night shifts, PSQI score continued to increase, but the rate of the increase gradually slowed. At about 12 years, PSQI scores leveled out toward a maximum.

**FIGURE 3:**
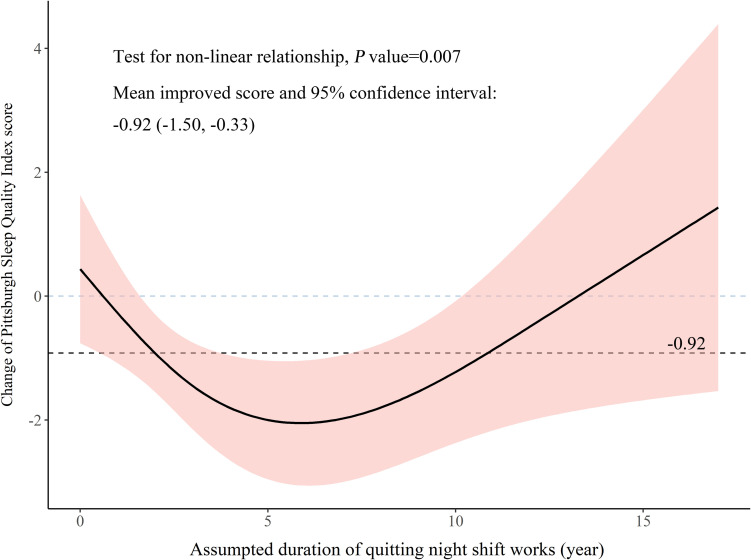
Change of sleep quality after quitting night shifts based on the hypothetical prospective cohort constructed from cross-sectional data.

**FIGURE 4:**
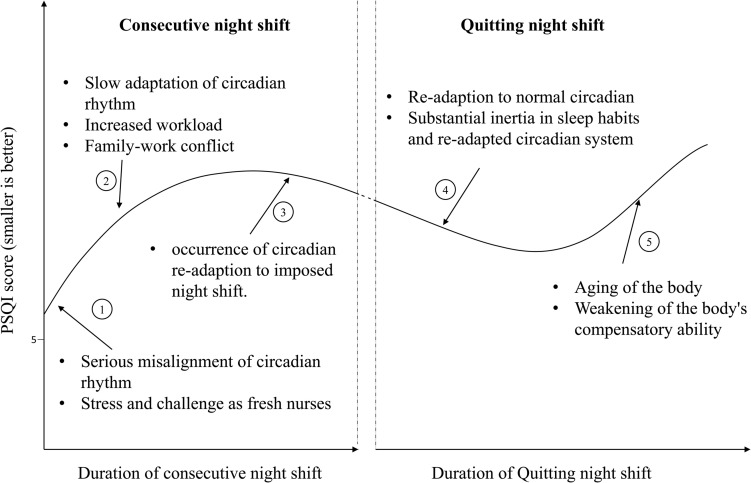
Possible reasons for change in Pittsburgh Sleep Quality Index (PSQI) during consecutive night shifts based on the cross-sectional data and change in PSQI after quitting night shifts based on the hypothetical prospective cohort.

In the hypothetical prospective cohort constructed from cross-sectional data, 98 pairs were created to explore the relationship between assumed duration of quitting night shift and change in PSQI. The mean difference in PSQI was −0.92 (95% CI: −1.50 to −0.33), the change was consistent with −0.91 (95% CI: −1.43 to −0.40) in 686 participants, which suggested that the constructed pairs were a representative subset of total participants. A U-shaped relationship between hypothetical years after quitting night shift and change in PSQI score was observed, *p* = 0.007, see Figure 3. After quitting night shift, change in PSQI score expanded gradually over about 6 years. Subsequently, the change reverted to 0. To present our results coherently and comprehensively, we connected the rising curve with a plateau and the U-shaped curve to form an S-shaped curve (Figure 4). Because the nurse might quit working consecutive night shifts at different times, the connection point of the rising curve and U-shaped curve could change. The S-shaped curve here was only used to explain the relationship.

## Discussion

In this study, poor sleep quality was reported in more than half of NCNS and NPNS; this was similar to results of other studies on similar subjects using PSQI > 5 as the cutoff (Kim et al., 2015; McDowall et al., 2017; Di Muzio et al., 2019). But the prevalence was higher than about 25% in the Chinese general population (Sun et al., 2015). In NCNS, we observed a rising relationship that plateaued at the end between PSQI score and years of consecutive night shifts. The mean PSQI score for those with few years of night shifts was greater than 5, and it leveled out toward a maximum at about 12 years. In the hypothetical cohort, a U-shaped relationship was observed in which the sleep quality was slightly improved after quitting night shifts but deteriorated again. It should be noted that the non-linear relationships in this cross-sectional study were exploratory analyses, and the results should be interpreted with caution.

The S-shaped curve documented the change in PSQI score; it was composed of an increasing curve during consecutive night shift years and a U-shaped curve after quitting night shifts. We divided the S-shaped curve into five segments (Figure 4). In segment 1, sleep quality deteriorated from the early stage of night shifts. The commencement of night shifts makes fresh nurses experience sudden misalignment of circadian rhythm and non-habitual sleep–wake schedule. Meanwhile, fresh nurses face numerous challenges and stress (Ebrahimi et al., 2016). Misalignment and stress led to poor sleep quality early in nursing careers. In segment 2, the PSQI score continued to rise. Adaptation of internal circadian clock to transitions between different shift schedules was slow (Arendt, 2010). The offshore-oil workers with 7 days of night shifts from 18:00 to 06:00 were studied, and the authors found a slow adaptation of 6-sulfatoxymelatonin (a circadian marker) but no improvement in objective sleep quality (Hansen et al., 2010). Most NCNS might have not adapted. As years of nursing work increased, NCNS would take more nursing responsibilities and get married. Work–family conflict may worsen sleep quality (Alhani and Mahmoodi-Shan, 2018). After about 12 years of consecutive night shifts, sleep quality seemed to be alleviated slightly, which formed segment 3. The alleviation might be due to the occurrence of adaptation to night shifts and work–family conflict.

After quitting night shifts, the sleep quality showed two-stage changes. In the first stage (segment 4), sleep quality improved slightly but did not return to normal. It suggested that the impact of night shift on sleep was alleviated; meanwhile, compensation for the long-term sleep disturbance might occur. However, previous night shift nurses reported that sleep problem did not disappear soon after quitting night shifts and the prevalence of some sleep problems was still high (Rotenberg et al., 2011). After a long-term night shift work, the nurses might have developed some sleep habits and adjusted their circadian rhythm to night shifts. However, the sleep practices that were adopted during night shift work might make it difficult to find quality sleep again during day shifts after quitting night shifts. In the second stage (segment 5), sleep quality deteriorated again. A 36-year follow-up study showed that the proportion of poor sleep increased over time (Hublin et al., 2018). The aging process decreases the resilience of circadian rhythm system, increases sensitivity to poor chrono-disruption, and weakens recovery ability, namely, sleep and circadian rhythm (SCR) frailty (Esquiva et al., 2017; Martinez-Nicolas et al., 2018). The change in SCR functions would increase the risk of SCR disturbance, which further led to sleep problems (Constance et al., 2016). We speculated that the decrease in sleep quality might result from the synergistic effects of the aging process and possible chronic sleep problems developed during long-term night shifts.

The duration of night shifts had a greater impact on health than the frequency of night shifts (Qiao et al., 2020). Two large cohort studies of female nurses found that the duration of night shifts and unhealthy lifestyle were independently and jointly associated with a higher risk of type 2 diabetes (Shan et al., 2018). Nursing managers can arrange for nurses to quit night shifts at an optimal time (for example, before sleep quality deteriorated to the peak or before it showed adaptation to night shifts). Sleep hygiene education programs should incorporate healthy lifestyle topics and can be accessible for all nurses (Sun et al., 2019). In addition, increasing the supply of fresh nurses and optimizing the allocation of nursing resources are effective methods to address the impact of night shifts.

### Limitations

Several limitations should be considered. Firstly, our study was limited by the nature of cross-sectional studies; the temporal relationship between the duration of nursing work with or without night shift and sleep quality cannot be estimated. Even though years of nursing work was adjusted, residual confounding of age was not excluded. A longitudinal repeated-measures study with normal control is needed to make causal inferences and estimate the lag effect. Secondly, PSQI was a standardized tool to quantify global sleep quality and disturbances among the general population; the single score might not be an ideal method for comprehensively assessing chronic sleep problems. In further research, other validated and professional measures (such as Insomnia Severity Index) and objective measures (such as actigraphy) can be used (Klingman et al., 2017). Thirdly, the sample size between the two groups was unbalanced; selection bias might be unavoidable. The study population was clearly identified, so realistic distribution of nursing resources and different numbers of nurses at different ages could explain the imbalance. In addition, a propensity score method was conducted to reduce the influence of selection bias. Lastly, the type and management of night shifts may be different in different countries and regions; the result in this study might not be generalizable to other countries. The non-linear change in PSQI could provide clues for future night shift-related studies. Meanwhile, a comparison between night shift nurses and other shift workers may provide further meaningful clues to improve management strategies of night shifts and lower the impact of night shifts.

## Conclusion

This study has shown that there was a high proportion of poor sleep quality in both NCNS and NPNS. During consecutive night shifts, sleep quality got worse gradually. During day shifts after quitting night shifts, poor sleep quality showed a slight but significant improvement; however, the U-shaped relationship indicated that improvement was not long-lasting. Nursing and hospital managers should arrange an appropriate time point for nurses to quit night shifts. To relieve the adverse effect of night shifts and poor sleep quality, sleep hygiene education should be provided early and regularly for all clinical nurses, and managerial and policy-based support should be increased.

## Data Availability Statement

The raw data supporting the conclusions of this article will be made available by the authors, without undue reservation.

## Ethics Statement

The studies involving human participants were reviewed and approved by the Ethics Committee of Tongji Medical College, Huazhong University of Science and Technology. The patients/participants provided their written informed consent to participate in this study.

## Author Contributions

CT and X-TZ have full access to all of the data in the study and take responsibility for the integrity of the data and the accuracy of the data analysis. QH and CT analyzed and interpreted the data of this study and drafted the manuscript. CT and X-TZ made critical revisions of the article for important intellectual content. X-TZ gave administrative, technical, or logistic support for conducting this study. All authors contributed to the article and approved the submitted version.

## Conflict of Interest

The authors declare that the research was conducted in the absence of any commercial or financial relationships that could be construed as a potential conflict of interest.

## Publisher’s Note

All claims expressed in this article are solely those of the authors and do not necessarily represent those of their affiliated organizations, or those of the publisher, the editors and the reviewers. Any product that may be evaluated in this article, or claim that may be made by its manufacturer, is not guaranteed or endorsed by the publisher.

## Abbreviations

PSQI: Pittsburgh Sleep Quality Index
NPNS: nurses who had worked Past Night Shifts
NCNS: Nurses who were on Consecutive Night Shifts
IDA Scale, Irritability: Depression and Anxiety Scale
PSS: Perceived Stress Scale
RCS: restricted cubic spline
SCR: sleep and circadian rhythms
VIF: variance inflation factor.
