# Supplementary material for: Poor Sleep Quality in Nurses Working or Having Worked Night Shifts: A Cross-Sectional Study
Source: Front Neurosci. 2021 Aug 3;15:638973. doi: 10.3389/fnins.2021.638973 (PMC8369413; doi:10.3389/fnins.2021.638973)
Supplement: Supplementary file 1 [file Table_1.DOCX]

**Table S1 Comparison of sleep quality, irritability, depression and anxiety, perceived stress between nurses with consecutive and past night shifts.**

| **Characteristics** | **Nurses with consecutive night shifts** | **Nurses with past**  **night shifts** | *P^a^* | *P^b^* |
| --- | --- | --- | --- | --- |
| Pittsburgh Sleep Quality Index (PSQI) | | | |  |
| PSQI global score, median(Q1, Q3) | 6.00(5.00, 8.00) | 6.00(4.00, 8.00) | - | 0.04 |
| Poor sleep quality, n(%)^c^ | 318(62.11%) | 97(55.75%) | 0.14 | - |
| Subjective sleep quality, n(%) |  |  | - | 0.03 |
| 0 | 32(6.25%) | 27(15.52%) |  |  |
| 1 | 316(61.72%) | 97(55.75%) |  |  |
| 2 | 148(28.91%) | 46(26.44%) |  |  |
| 3 | 16(3.13%) | 4(2.30%) |  |  |
| Sleep latency, n(%) |  |  | - | 0.04 |
| 0 | 78(15.23%) | 46(26.44%) |  |  |
| 1 | 214(41.80%) | 56(32.18%) |  |  |
| 2 | 138(26.95%) | 55(31.61%) |  |  |
| 3 | 82(16.02%) | 17(9.77%) |  |  |
| Sleep duration, n(%) |  |  | - | 0.22 |
| 0 | 95(18.55%) | 42(24.14%) |  |  |
| 1 | 381(74.41%) | 119(68.39%) |  |  |
| 2 | 30(5.86%) | 10(5.75%) |  |  |
| 3 | 6(1.17%) | 3(1.72%) |  |  |
| Habitual sleep efficiency, n(%) |  |  | - | 0.15 |
| 0 | 368(71.88%) | 136(78.16%) |  |  |
| 1 | 103(20.12%) | 23(13.22%) |  |  |
| 2 | 27(5.27%) | 11(6.32%) |  |  |
| 3 | 14(2.73%) | 4(2.30%) |  |  |
| Sleep disturbance, n(%) |  |  | - | 0.49 |
| 0 | 23(4.49%) | 12(6.90%) |  |  |
| 1 | 394(76.95%) | 121(69.54%) |  |  |
| 2 | 91(17.77%) | 41(23.56%) |  |  |
| 3 | 4(0.78%) | 0(0.00%) |  |  |
| Use of sleep medication, n(%) |  |  | - | 0.03 |
| 0 | 478(93.36%) | 153(87.93%) |  |  |
| 1 | 24(4.69%) | 17(9.77%) |  |  |
| 2 | 6(1.17%) | 2(1.15%) |  |  |
| 3 | 4(0.78%) | 2(1.15%) |  |  |
| Daytime dysfunction, n(%) |  |  | - | 0.65 |
| 1 | 340(66.41%) | 119(68.39%) |  |  |
| 2 | 155(30.27%) | 49(28.16%) |  |  |
| 3 | 17(3.32%) | 6(3.45%) |  |  |
| Irritability, Depression and Anxiety Scale (IDA) , median(Q1, Q3) | | | | |
| a. Depression score | 5.00(3.00, 7.00) | 5.00(4.00, 7.00) | - | 0.53 |
| b. Anxiety score | 6.00(4.00, 8.00) | 6.00(4.00, 8.00) | - | 0.27 |
| c. Inward irritability | 4.00(2.00, 5.00) | 4.00(2.00, 6.00) | - | 0.71 |
| d. Outward irritability | 4.00(3.00, 6.00) | 4.00(3.00, 6.00) | - | 0.97 |
| Perceive stress scale (PSS) , median(Q1, Q3) | | | | |
| Perceived stress score | 25.00(20.00, 29.00) | 26.00(21.00, 30.00) | - | 0.28 |
| a. self-efficacy | 13.00(10.00, 17.00) | 13.50(10.00, 17.00) | - | 0.72 |
| b. perceived helplessness | 11.00(9.00, 14.00) | 12.00(10.00, 15.00) | - | 0.07 |

^a^ *p* value from chi-square test. ^b^ *p* value from Wilcoxon rank-sum test. ^c^ PSQI score>5.
